# SUBCLINICAL AGE-RELATED HEARING LOSS IS INVERSELY ASSOCIATED WITH DEPRESSIVE SYMPTOMS

**DOI:** 10.1093/geroni/igz038.2034

**Published:** 2019-11-08

**Authors:** Justin S Golub, Katharine K Brewster, Adam Brickman, Adam Ciarleglio, José Luchsinger, Bret Rutherford

**Affiliations:** 1 Columbia University, New York, New York, United States; 2 Columbia University Medical Center, New York, New York, United States; 3 George Washington University, Washington, District of Columbia, United States

## Abstract

Age-related hearing loss (HL), defined by a pure-tone average (PTA) >25 decibels (dB) has been associated with depressive symptoms. We aimed to assess whether this association is present when hearing is better than the arbitrary, but widely-used, 25 dB threshold. The sampled population was the multicentered Hispanic Community Health Study (n=5,165). Cross-sectional data from 2008-2011 were available. Hearing was measured with pure tone audiometry. Clinically-significant depressive symptoms (CSDS) were defined by a score ≥10 on the 10-item Center for Epidemiologic Studies Depression Scale (CESD-10). Participants’ mean age was 58.3 years (SD=6.2, range=50-76). Among those with classically-defined normal hearing (PTA ≤25 dB), a 10 dB increase in HL was associated with 1.26 times the odds (95% CI=1.11, 1.42) of CSDS, adjusting for age, gender, education, vascular disease, and hearing aid use (p25 dB; p<0.001). Results held even for a stricter HL cutpoint of 15 dB. Among subjects with strictly normal hearing (PTA ≤15 dB), a 10 dB increase in HL was associated with 1.47 (1.14, 1.90) times the odds of CSDS, adjusting for confounders (p<0.01). Results also held when defining CSDS by an alternative CESD-10 score ≥16. In conclusion, increasing hearing thresholds were independently associated with CSDS among adults with subclinical HL (PTA ≤25 dB). Studies investigating whether treating HL can prevent late life depression should consider a lower threshold for defining HL.

